# A Novel Integrated Structure with a Radial Displacement Sensor and a Permanent Magnet Biased Radial Magnetic Bearing

**DOI:** 10.3390/s140201950

**Published:** 2014-01-24

**Authors:** Jinji Sun, Yin Zhang

**Affiliations:** School of Instrumentation Science and Optoelectronics Engineering, Beihang University, Beijing 100191, China; E-Mail: heihuo1986@gmail.com

**Keywords:** displacement sensor, integrated structure, magnetic bearing, permanent magnet biased

## Abstract

In this paper, a novel integrated structure is proposed in order to reduce the axial length of the high speed of a magnetically suspended motor (HSMSM) to ensure the maximum speed, which combines radial displacement sensor probes and the permanent magnet biased radial magnetic bearing in HSMSM. The sensor probes are integrated in the magnetic bearing, and the sensor preamplifiers are placed in the control system of the HSMSM, separate from the sensor probes. The proposed integrated structure can save space in HSMSMs, improve the working frequency, reduce the influence of temperature on the sensor circuit, and improve the stability of HSMSMs.

## 1. Introduction

Magnetic bearings have some advantages such as no mechanical friction, no wear, no lubrication, long life, and high reliability, therefore, they can be applied in flywheels [1], air compressors, molecular pumps, turbines, generators, and bearingless motors [2–5]. To decrease the power losses of magnetic bearings, the permanent magnet biased magnetic bearing, which is also called hybrid magnetic bearing, is adopted widely [6–9]. In magnetic bearing systems, displacement sensors, which can detect the rotor's displacement in five degrees of freedom (DOF) along the corresponding direction, are necessary. The most frequently used displacement sensors are the eddy current sensors since they have high resolution and wide bandwidth in active magnetic bearing systems [10–13]. This type of sensor is very easily influenced by the magnetic field generated in magnetic bearing coils and so should be installed outside the coils [14]. Consequently, radial displacement sensors are designed to separate from the radial magnetic bearings in general, and a large axial length will be used, so the rotor modal shape is low, and bending vibrations of the rotor will be produced, resulting in a lower maximum high speed of the magnetically suspended motor (HSMSM).

In addition, eddy current sensors mainly include preamplifier circuits and probes, which are integrated in the HSMSM. As a result, the performance of electronic components such as amplifiers, resistors, capacitors and diodes of the preamplifier circuit are easily affected by temperature, and they easily burn in the usual environment of vibrations and moisture. Therefore, existing eddy current sensors are not good with respect to compactness, environmental adaptability and so on.

In this paper, a novel integrated structure is proposed, which combines the radial displacement sensor with eddy current and the radial magnetic bearing with permanent magnet bias. It takes advantage of the space in the radial magnetic bearing, and the sensor probes are placed on the adjacent stator poles in the axial direction. The preamplifier circuit is placed outside the HSMSM and uses shielded cables to connect the sensor probes. Therefore, the axial size of the HSMSM can be effectively reduced and the modal shape of the rotor can be increased. More importantly, the preamplifier circuit is not directly affected by the temperature. The prototype integrated structure was manufactured and verified by experimental tests, and the performance of the sensor is improved in aspects such as the accuracy of detecting displacements, linearity and temperature drift.

## 2. Structure

**Figure 1.**
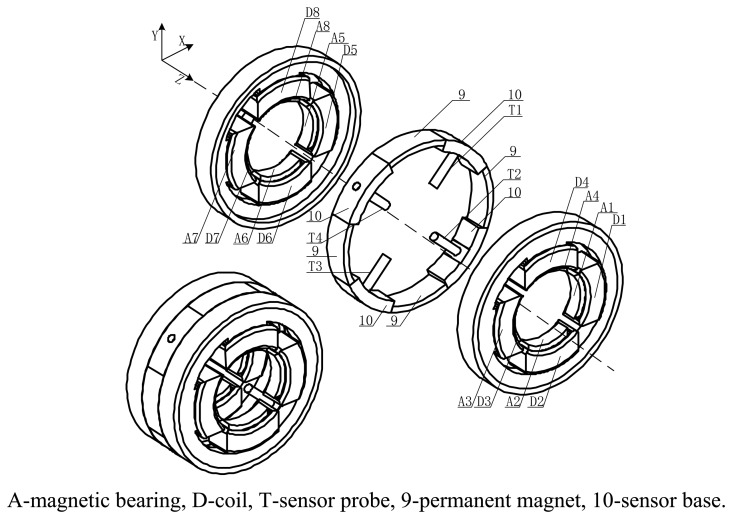
Three-dimensional diagram and exploded view of the proposed novel structure.

**Figure 2.**
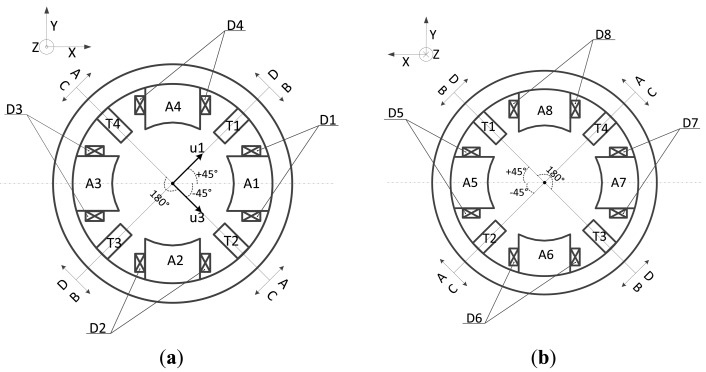
The novel proposed structure: (**a**) Front view; (**b**) Rear view.

The proposed novel structure is composed of displacement sensor probes, a permanent magnet biased radial magnetic bearing and an external signal processing circuit. A three-dimensional diagram and exploded view of the proposed novel structure is shown in Figure 1 and the front view and the rear view of the integrated structure are shown in Figure 2, respectively. In these figures, it can be seen that the radial magnetic bearing consists of magnetic poles (A1∼A8), coils (D1∼D8), and permanent magnets (9). The magnetic poles have eight and are placed in the X and Y directions, in two groups along the Z direction (A1∼A4 and A5∼A8). Each group of magnetic poles contains four separated by 90 degrees along the circumference in the +x, −x, +y and −y directions. In the figure, magnetic poles (A1, A3, A5, A7) are placed in the x direction and form a group, and magnetic poles (A2, A4, A6, A8) are placed in the y direction and form another group. There are four permanent magnets (9) and four sensor bases (10), and discrete permanent magnets and sensor bases are mounted between two groups of magnetic poles. Permanent magnets and sensor bases are arranged alternately along the circumference. That is to say, the discrete permanent magnets are placed along the circumference in the x and y directions, and sensor bases are installed among the adjacent permanent magnets. The sensor bases are made of aluminum and sensor probes (T1∼T4), which are uniformly distributed along circumference, are amounted on them with glue. The rotor is suspended stably by control currents in every coil wound on the corresponding magnetic poles.

**Figure 3.**
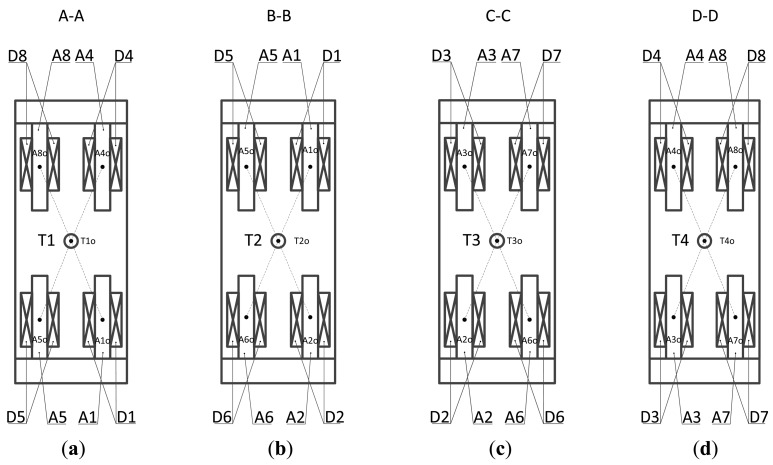
Various views of the novel proposed structure: (**a**) Section A-A; (**b**) Section B-B; (**c**) Section C-C; (**d**) Section D-D.

In Figure 3 various views of the novel proposed structure are given. It can be seen that the distance between the central position T1_0_ of sensor probe T1 and the central position A1_0_ of the magnetic pole is equal to the distance between T1_0_ and A4_0_, as well as the distance between T1_0_ and A5_0_ and the distance between T1_o_ and A8_o_, as shown in Figure 3a. Likewise, the distances are equal among the central position of the sensor probe and other corresponding central points of the magnetic poles. For instance, L_T2o, A1o_ = L_T2o, A2o_ = L_T2o, A5o_ = L_T2o, A6o_, L_T3o, A2o_ = L_T3o, A3o_ = L_T3o, A6o_ = L_T3o, A7o_, and L_T4o, A3o_ = L_T4o, A4o_ = L_T4o, A7o_ = L_T4o, A8o_, as shown in Figure 3b–d.

**Figure 4.**
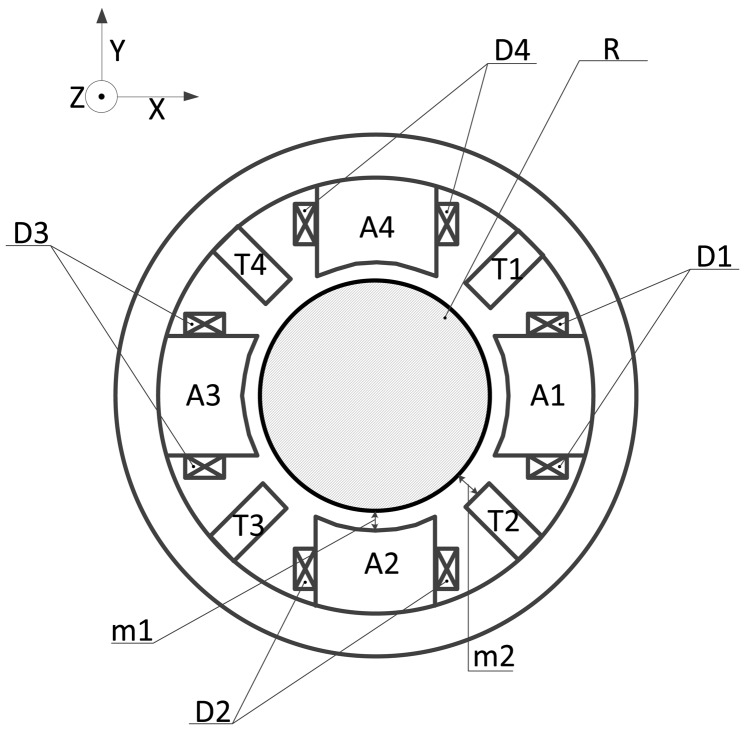
The main view of the proposed novel structure, including the HSMSM rotor.

In practical fact, a pair of differential output probes is composed by sensor probes (T1, T3) as well as the other sensor probes (T2, T4). As shown in Figure 4, an air gap is formed between magnetic poles (A1∼A8) and rotor (R) and the detection gap is formed between sensor probes (T1∼T4) and rotor (R). The length of the air gap (m1, m2) is designed to be 0.4∼0.5 mm and the detection gap is designed to be 0.75∼1.25 mm.

**Figure 5.**
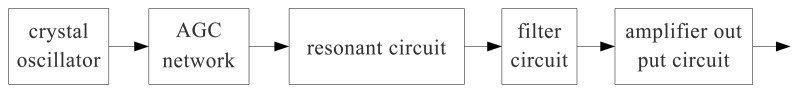
The diagram of the preamplifier.

As shown in Figure 5, the displacement sensor probe (T1∼T4) is mainly composed of a crystal oscillator, AGC network, resonant circuit, filter circuit and amplifier output circuit. The crystal oscillator is used to provide a stable frequency and amplitude for the excitation signal.

**Figure 6.**
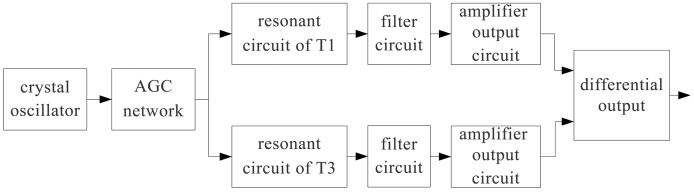
The compensation circuit principle diagram of each pair of preamplifiers.

As shown in Figure 6, a differential structure is formed between the preamplifiers of the displacement sensor probes T1 and T3 as well as T2 and T4, that is to say, the circuit structures of the preamplifiers are identical and symmetrical. The resonant circuits of sensor T1 and T3 are the same as sensor T2 and T4 as well. The differential structures can restrain the temperature drift and time drift, and improve the sensor's temperature and time stability.

As we know, the principle of the eddy current displacement sensor is the mutual inductance effect between a high frequency current in coils and detector. Therefore, the detector material has an important influence on the sensitivity and precision of the displacement sensor. Steels such as 45# or 40Cr can often be used considering their stability.

## 3. Magnetic Field Analysis

**Figure 7.**
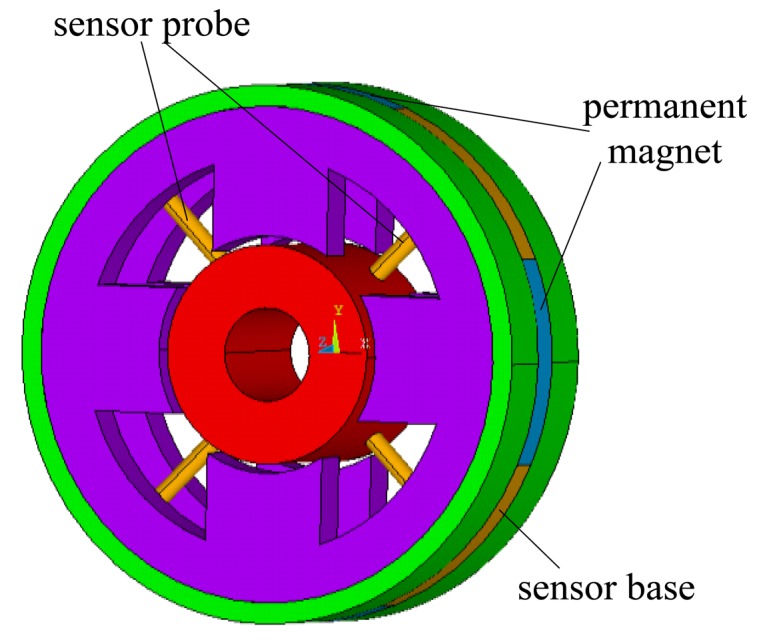
3D FEM model of the proposed structure.

**Figure 8.**
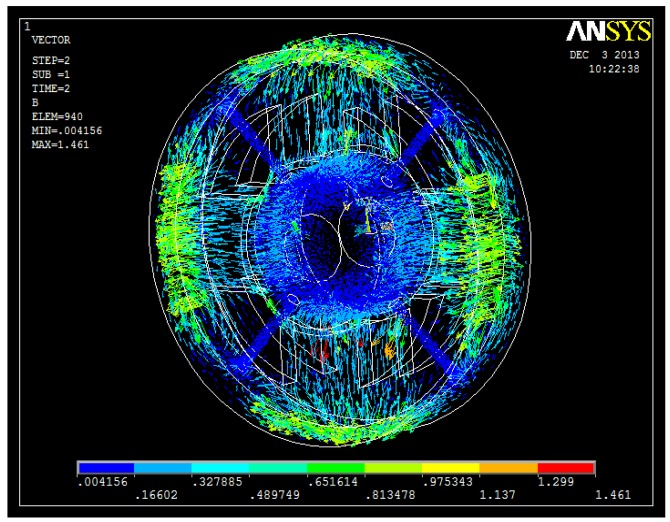
Flux distribution of the proposed structure.

**Figure 9.**
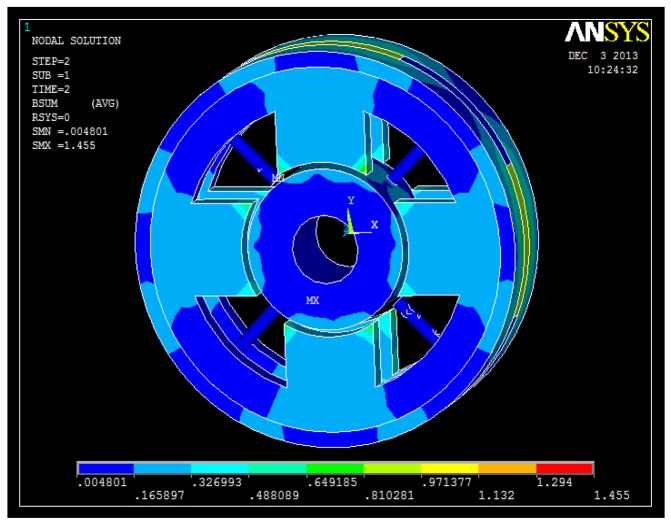
Flux density distribution of the proposed structure.

**Figure 10.**
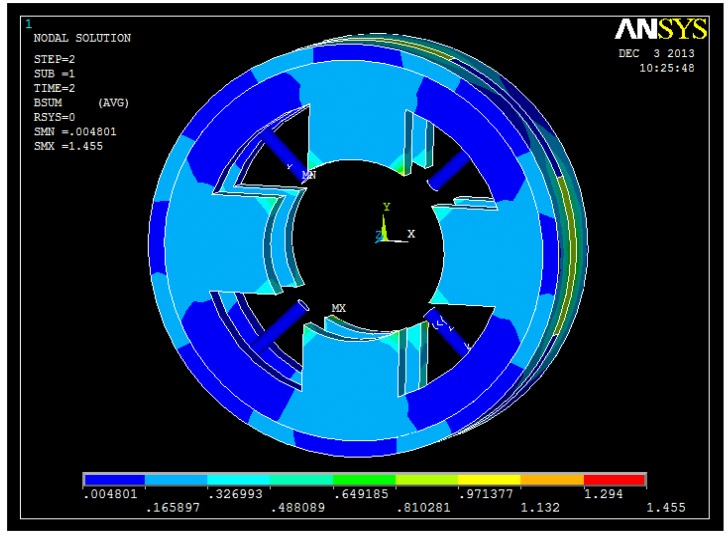
Flux density distribution of the stator in the proposed structure.

The 3D FEM analyses are shown in Figures 7, 8, 9 and 10. From these figures, we can see that the magnetic field is weak at the sensor probes, so we can conclude that the sensor probes will not be influenced by the magnetic field produced by the radial magnetic bearing.

## 4. Experimental Test

**Figure 11.**
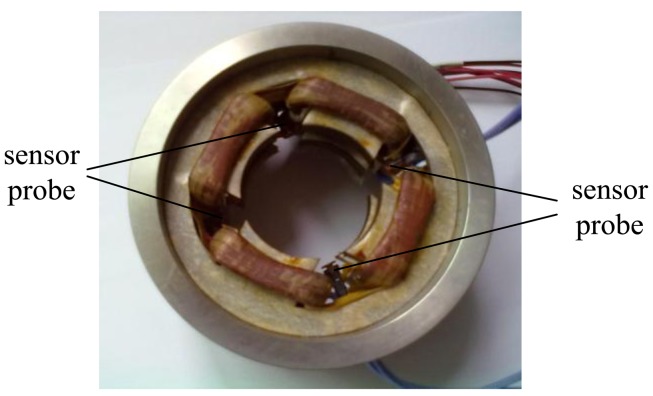
Photograph of the proposed structure with the radial displacement sensors and radial magnetic bearing.

**Figure 12.**
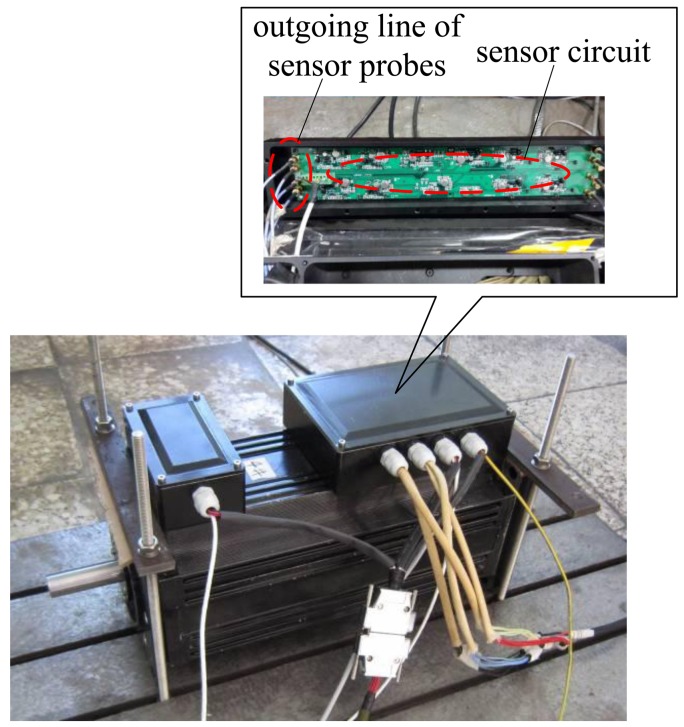
Photograph of a HSMSM with the proposed structure.

The photograph of the proposed novel structure is shown in Figure 11, and it is seen clearly that the four sensor probes are placed between two groups of stator magnetic poles, which are distributed uniformly along the circumstance, so the radial magnetic bearing is integrated with the radial sensor probes. Figure 12 shows a photograph of a 4 kW HSMSM with the proposed structure, and it can be seen that the sensor circuit is separate from the sensor probes. The whole axial length can be reduced by 9% compared to the structure in which the radial magnetic bearing is separated from the radial sensor probe by calculation, and then the first bending mode will increase about 16%, as well as the maximum critical speed.

**Figure 13.**
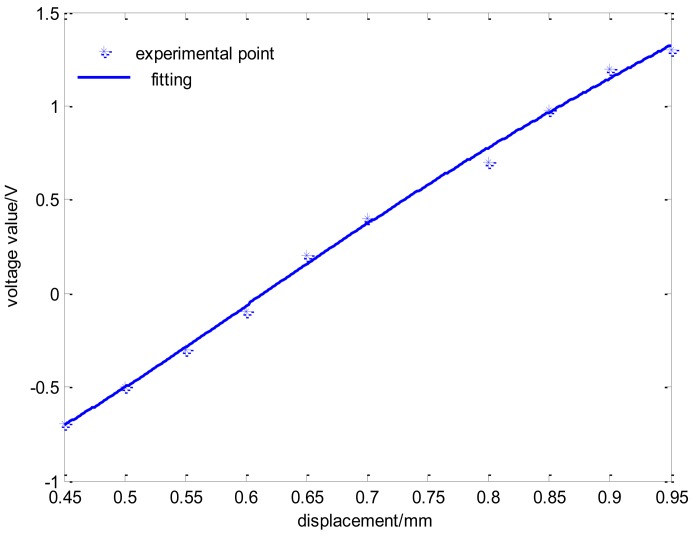
The relationship between displacement and output voltage value.

**Figure 14.**
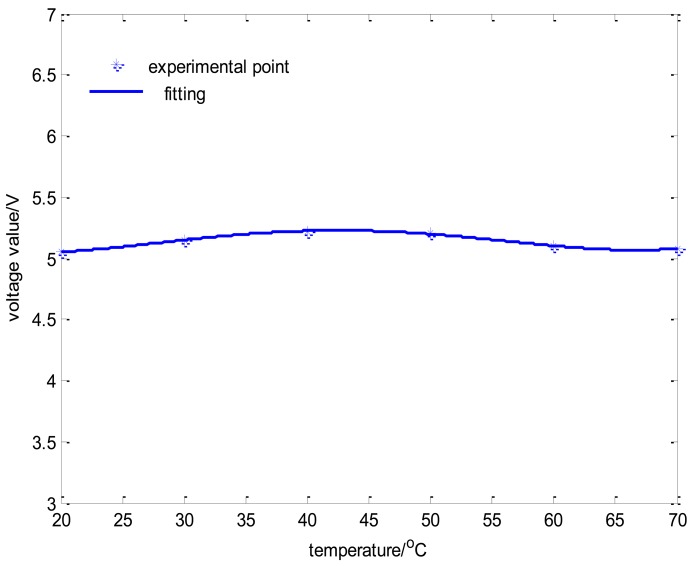
The relationship between temperature and output voltage value.

The relation between displacement and output voltage is shown in Figure 13, and it is indicated that the curve has a good linearity in order to meet the sensor demands. In addition, the relationship between the temperature of the sensor circuit and output voltage is shown in Figure 14, where the output voltage ripple is approximately 4% as the temperature of the sensor circuit in the offline experiment varies from 20 °C to 70 °C. The displacement curve cannot reflect the change of temperature drift because it always remains constant under the condition of closed loop control. Therefore, the coil current curves of every channel can reflect the temperature drift effectively.

**Figure 15.**
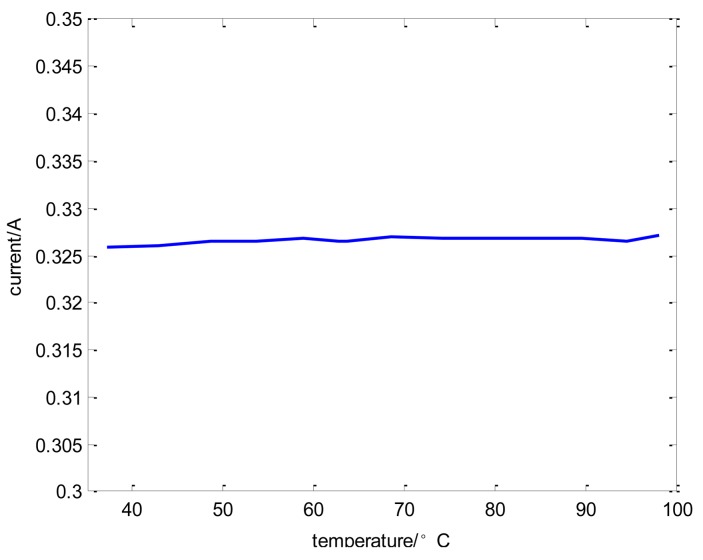
The relationship between temperature and coil current.

**Figure 16.**
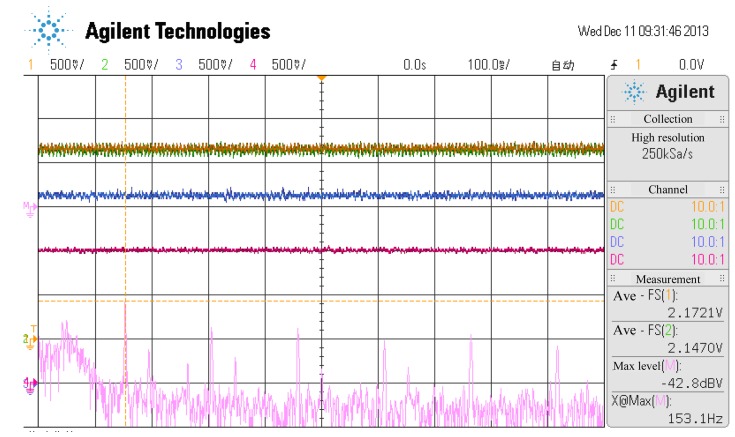
The current curves of every channel when the temperature of the sensor probe is at 37 °C.

**Figure 17.**
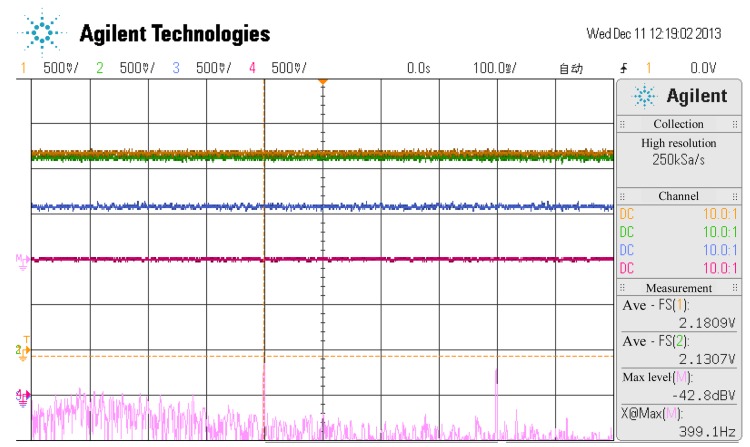
The current curves of every channel when the temperature of the sensor probe is at 98 °C.

The relationship between the temperature of the sensor probe and the coil current of a channel is shown in the online experiment in Figures 15, 16 and 17 where the temperature of the sensor probe is at 37 °C and 98 °C, respectively. In these figures, 1 V denotes 0.15 A. To be sure, the sensor circuit is separated from sensor probes, so the temperature of the sensor circuit is from 20 °C to 70 °C, while the temperature of the sensor probes is from 37 °C and 98 °C.

## 5. Conclusions

A novel integrated structure to reduce the axial length and save space in a HSMSM is proposed, which combines a radial displacement sensor and a radial magnetic bearing. The whole axial length can be reduced 9% in a 4 kW HSMSM system, and the first bending mode will increase about 16%, as well as the maximum critical speed. The presented integrated structure separates the sensor probes from their circuit. Through an analysis of the magnetic field, it is determined that it is weak at the sensor probes, so they are not be influenced by the magnetic field produced by the radial magnetic bearing. A prototype of the proposed integrated structure is manufactured and it has good linear displacement characteristics as well as temperature characteristics according to experimental tests.
